# Implicit Memory in Monkeys: Development of a Delay Eyeblink Conditioning System with Parallel Electromyographic and High-Speed Video Measurements

**DOI:** 10.1371/journal.pone.0129828

**Published:** 2015-06-12

**Authors:** Yasushi Kishimoto, Shigeyuki Yamamoto, Kazutaka Suzuki, Haruyoshi Toyoda, Masanobu Kano, Hideo Tsukada, Yutaka Kirino

**Affiliations:** 1 Department of Neurobiophysics, Kagawa School of Pharmaceutical Sciences, Tokushima Bunri University, Sanuki, Kagawa, Japan; 2 Central Research Laboratory, Hamamatsu Photonics K.K., Hamakita-ku, Hamamatsu, Japan; 3 Department of Neurophysiology, Graduate School of Medicine, University of Tokyo, Bunkyo-ku, Tokyo, Japan; University of Toyama, JAPAN

## Abstract

Delay eyeblink conditioning, a cerebellum-dependent learning paradigm, has been applied to various mammalian species but not yet to monkeys. We therefore developed an accurate measuring system that we believe is the first system suitable for delay eyeblink conditioning in a monkey species (*Macaca mulatta*). Monkey eyeblinking was simultaneously monitored by orbicularis oculi electromyographic (OO-EMG) measurements and a high-speed camera-based tracking system built around a 1-kHz CMOS image sensor. A 1-kHz tone was the conditioned stimulus (CS), while an air puff (0.02 MPa) was the unconditioned stimulus. EMG analysis showed that the monkeys exhibited a conditioned response (CR) incidence of more than 60% of trials during the 5-day acquisition phase and an extinguished CR during the 2-day extinction phase. The camera system yielded similar results. Hence, we conclude that both methods are effective in evaluating monkey eyeblink conditioning. This system incorporating two different measuring principles enabled us to elucidate the relationship between the actual presence of eyelid closure and OO-EMG activity. An interesting finding permitted by the new system was that the monkeys frequently exhibited obvious CRs even when they produced visible facial signs of drowsiness or microsleep. Indeed, the probability of observing a CR in a given trial was not influenced by whether the monkeys closed their eyelids just before CS onset, suggesting that this memory could be expressed independently of wakefulness. This work presents a novel system for cognitive assessment in monkeys that will be useful for elucidating the neural mechanisms of implicit learning in nonhuman primates.

## Introduction

Classical eyeblink conditioning is one of the best-characterized behavioral models of associative learning in mammals [1, 2]. In this type of learning task, after repeated presentation of a conditioned stimulus (CS, e.g., tone) paired with an unconditioned stimulus (US, e.g., air puff), subjects learn to close the eye before onset of the US so that the eye is closed when the air puff is delivered. Most eyeblink conditioning can be classified into two distinct types: delay and trace conditioning [1, 3]. The standard delay paradigm in which the CS and US are co-terminal in time but the CS starts earlier is cerebellum-dependent and is supposed to be unrelated to awareness in humans [4–8]. This paradigm has been used extensively for assessing motor learning or implicit memory performance in a variety of mammalian species including human, cat, rabbit, rat, and mouse [9–14], as well as reptiles [15]. The delay version of eyeblink conditioning in monkeys has not yet been reported although trace eyeblink conditioning in monkeys was investigated using an infrared camera [16]. However, no attempt has been made so far to evaluate eyeblink conditioning using electromyographic (EMG) methods or video monitoring, which are the de facto standard methods for evaluating eyelid response activities during eyeblink conditioning in many species including humans and rodents [9, 10, 16–19].

Hence, the primary aims of the present study were three-fold: first, to examine whether delay eyeblink conditioning using an EMG measuring method is possible in monkeys; second, to do the same for a high-speed (1 kHz) camera-based tracking system [20] for eyeblink detection, which has a higher spatial-time resolution than the infrared camera method [16]; and third, in the case of successful monkey delay conditioning, to confirm the usefulness of the two methods for evaluating conditioned responses (CRs). We also wanted to compare and contrast the sensitivities of the two methods for detecting CR incidence and timing in delay eyeblink conditioning in monkeys.

Here, we report a new multi-measuring system for analyzing monkey eyeblink conditioning using simultaneous EMG analysis and high-speed video analysis. Both methods could sensitively detect eyeblink CRs during acquisition and extinction sessions in monkeys. This multi-measuring system incorporating two different measuring principles enabled us to elucidate the relationship between the actual state of eyelid closure and OO-EMG activities during the experiment. The characteristics of our experimental method led to an interesting finding—that the monkeys frequently exhibited obvious CRs even when their eyes were nearly closed.

## Materials and Methods

### Animals

Rhesus monkeys (*Macaca mulatta*) were obtained from Hamri Company, Ltd. (Ibaraki, Japan). Five male monkeys (4–5 years of age, each weighing 4.0–5.0 kg) were used in this study. All experiments were performed during the light phase of a 12-hour light-dark cycle. This study was conducted in strict accordance with the recommendations in the Guide for the Care and Use of Laboratory Animals of the National Institutes of Health. The complete study was approved by the Animal Care and Use Committee of Hamamatsu Photonics K.K. All surgery was performed under sodium pentobarbital anesthesia, and all efforts were made to minimize suffering. The monkeys were housed in individual primate cages with controlled humidity, temperature, and environmental enrichment (toys). They had auditory, visual, and olfactory contact with other monkeys. Food and water were available ad libitum at the home cage after each training. Additionally, the monkeys were fed a variety of vegetables and fruits every day. To assess the monkeys' health, their weight was routinely monitored and expert veterinarian assistance was available on site. Animals were not sacrificed after the experiments.

### Conditioning procedures

#### Habituation

**Fig 1:**
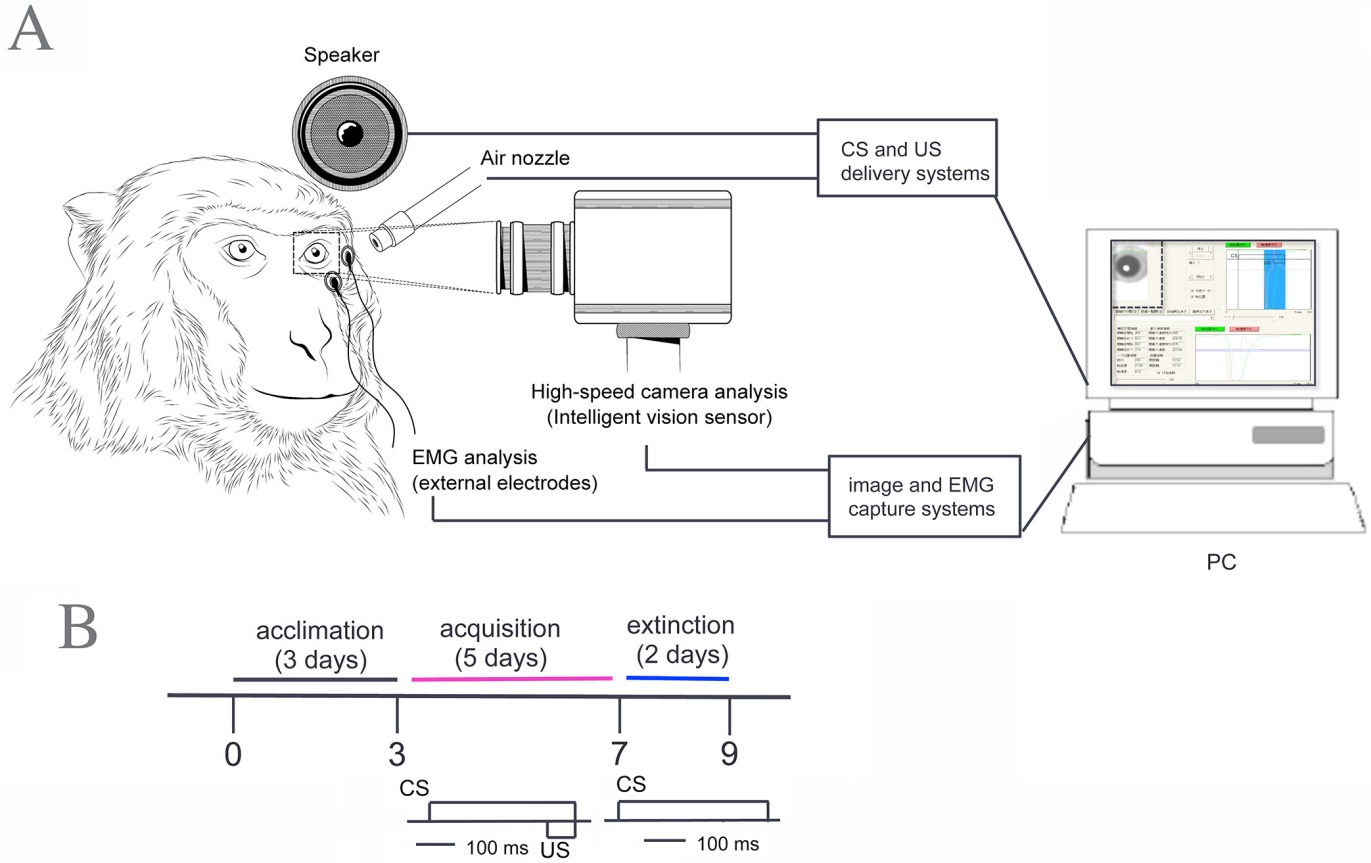
Schematic diagram of the system for evaluating eyeblink conditioning in monkeys. (A) The multi-measuring system for detecting delay eyeblink conditioned responses (CRs) in monkeys is illustrated. The system consists of PC-based software and hardware that control the experiment by activating the conditioned stimulus (CS) and unconditioned stimulus (US) delivery systems and that acquire electromyographic (EMG) and video data from skin electrodes and a digital camera, respectively. A monkey is shown restrained as it appears during eyeblink conditioning. The eyeblink behavior of the monkey is simultaneously measured and detected by EMG and video. During conditioning, a 1-kHz tone from a speaker close to the left ear provides the CS. The US is an air-puff delivered from a nozzle to the left cornea. EMG signals are picked up from the left temple, and the movement of the left eyelid (ipsilateral to the air-puff stimulation) is measured by the Intelligent Vision Sensor. *Inset upper right*: the temporal relationship between the CS and US in the delay eyeblink conditioning paradigm. (B) The experimental design for delay eyeblink conditioning in monkeys. The moment of the CS and US presentations is shown under the timeline for behavioral test. See Materials and Methods for details.

An acrylic head holder (4.5 cm width × 5.5 cm length × 3.5 cm height) was attached to the top of each monkey’s skull under pentobarbital anesthesia [21]. The head holder was used for painless fixation of the monkey’s head during the measurement of eyeblink responses. The acrylic head holder was not removed from the monkeys' skull at the end of the experiment. Monkeys were not noticeably disturbed by the acrylic head holder because the head holder was small and light. The monkeys moved freely in their cages and ate food without any difficulties as they did before surgery. The monkeys will be re-used in future experiments. After the monkey had been placed in the monkey chair, it was transferred to a measurement room equipped with the recording and stimulation devices. After habituation to the monkey chair and room for 2 hours a day for 1 month, monkeys completed the delay eyeblink conditioning paradigm in which the CS begins before and co-terminates with the US (inset in Fig 1A). Fig 1B illustrates an experimental schedule for the conditioning.

#### Apparatus

The system configuration comprised of four parts (Fig 1A): a restraining device, recording apparatus, stimulation apparatus, and other analysis equipment. To detect and characterize eyeblink behavior with high temporal resolution, we adopted the EMG technique (sampling rate = 1 kHz) and used a high-speed camera-based tracking system capable of image capturing, processing, and control-signal output with 1-ms precision [20]. This system enabled us to monitor and detect eyeblink in a monkey using simultaneous orbicularis oculi electromyographic (OO-EMG) measurements and video eyelid-state detection. Our custom-built apparatus consisted of an air compressor (Kuroda, Japan), air regulator, solenoid valve, amplifier (Nihon Kohden, Japan), and personal computer (Dell, USA) in which LabVIEW software (National Instruments, USA) was installed. The conditioned stimulus (CS) was a tone generated by a speaker. The unconditioned stimulus (US) was an air puff supplied through a plastic tube from a pressure regulator as described in a previous study [16]. The personal computer was used to regulate the timing of the tones and air puffs. When a controlling solenoid valve opened, an air puff was directed at an upper eyelid line via an acrylic tube (4 mm in diameter, 1 m long). The end of the plastic tube was connected to a stainless steel tube (3 mm in diameter) that was fixed to the monkey chair. An orbicularis oculi response was measured using two Ag/AgCl_2_ electrodes (5 mm diameter) fixed to the skin over the left orbicular muscle of the eye. Hair over the orbicular muscle was shaved, and skin conductance was under 15 kΩ. One electrode was 5 mm lateral to the eye, and the other was 10 mm below the eye. The interelectrode distance was 12 mm. Both electrodes were equidistant from the center of the left eye. In addition, a reference electrode was placed behind the left ear over the mastoid. EMG signals were amplified (MEG-2100, Nihon Kohden), band-pass filtered (-3 dB high-pass cutoff at 1.5 Hz; -3 dB low-pass cutoff at 1 kHz), and digitized with a sampling rate of 1 kHz.

#### Conditioning procedure

A 350-ms tone (1 kHz, 85 dB) was used as the CS, and a 100-ms air-puff (0.02 MPa) was used as the US. Each session consisted of 100 trials grouped into 10 blocks of 10. Each session consisted of 10 pure CS presentations (every tenth trial in a block), 10 pure US presentations (every fifth trial in a block), and 80 CS-US paired trials. The intertrial interval was randomized between 15 and 25 s with a mean of 20 s. In delay conditioning with CS-US paired trials, the US was delivered after the start of the longer CS, and the two stimuli terminated simultaneously. Monkeys received seven sessions in total, comprising five acquisition sessions followed by two extinction sessions (one session per day). During an extinction session, 100 pure CS presentations were given.

### EMG analysis of eyelid closure

The EMG picked up from the left temple was analyzed by custom software as described previously [22–25]. The data for each session were processed off-line as follows: (i) The maximum amplitude of the EMG signal during a time period of t ± 1 ms was calculated and denoted the EMG amplitude at t. (ii) The 100 EMG amplitude values in the 300-ms period before CS onset were averaged, and the standard deviation (SD) was calculated. (iii) The average value obtained from (ii) + SD was defined as the threshold. (iv) In each trial, the EMG amplitude data from the 300-ms period before CS onset that were over the threshold were averaged and called the pre value. The startle value (alpha response) was calculated in the same way for the 50 ms following CS onset. The CR value was calculated from the data recorded in the period 0–200 ms before the US onset in the CS-US paired trials. The time window was extended by 100 ms to obtain the CR value in the CS-only trials. (v) Valid trials were defined as those with pre and startle values of less than 10% and 30% of the threshold, respectively. (vi) A trial in which the CR value exceeded 1% of the threshold and exceeded twice the pre value was regarded as a successful CR trial. (vii) The ratio of successful CR trials to valid trials was calculated and denoted the CR incidence (CR%).

### High-speed video-based tracking analysis for eyelid closure

The high-speed and high-accuracy measurement of eyeblink employed a high-speed CMOS image sensor (Intelligent Vision Sensor, C8201; Hamamatsu Photonics K.K.) capable of image capturing and processing at 1000 frames per second [26]. The procedure was similar to that used previously with human subjects [27, 28]. Briefly, eyelid movements were recorded with a CMOS image sensor-based camera with a time resolution of 1 ms. The upper lid position was extracted by the image-processing unit, and the velocity was calculated as the difference in the upper eyelid positions between two successive images. This processing comprised the following: i) calculation of horizontal projection data of image intensity, ii) determination of upper eyelid position as the point midway between the darkest area (pupil region) and brightest area (skin of the upper eyelid) on the projection data, and iii) evaluation of blink duration and extent in every conditioning trial in the same time windows as those used in the EMG analysis. However, the eyelid distance metric was defined as the distance between the upper and lower eyelids, and a naturally closed eye was defined as having a distance of zero. The occurrence of an eyelid response was determined by evaluating the acceleration and distance of the upper eyelid [27]. In the video analysis, we further identified the animal’s visible signs of drowsiness and behavioral microsleep by distinguishing among fully closed, half closed, and fully open eyes [29].

### Statistical analysis

Data were statistically analyzed with two-tailed Student’s *t*-tests using Microsoft Excel (USA) or with a repeated-measures analyses of variance (ANOVAs) followed by post hoc Scheffé testing using the SPSS 6.1 software (USA). Differences were considered significant at *p* < 0.05.

## Results

### Delay eyeblink conditioning in monkeys by EMG analysis

**Fig 2:**
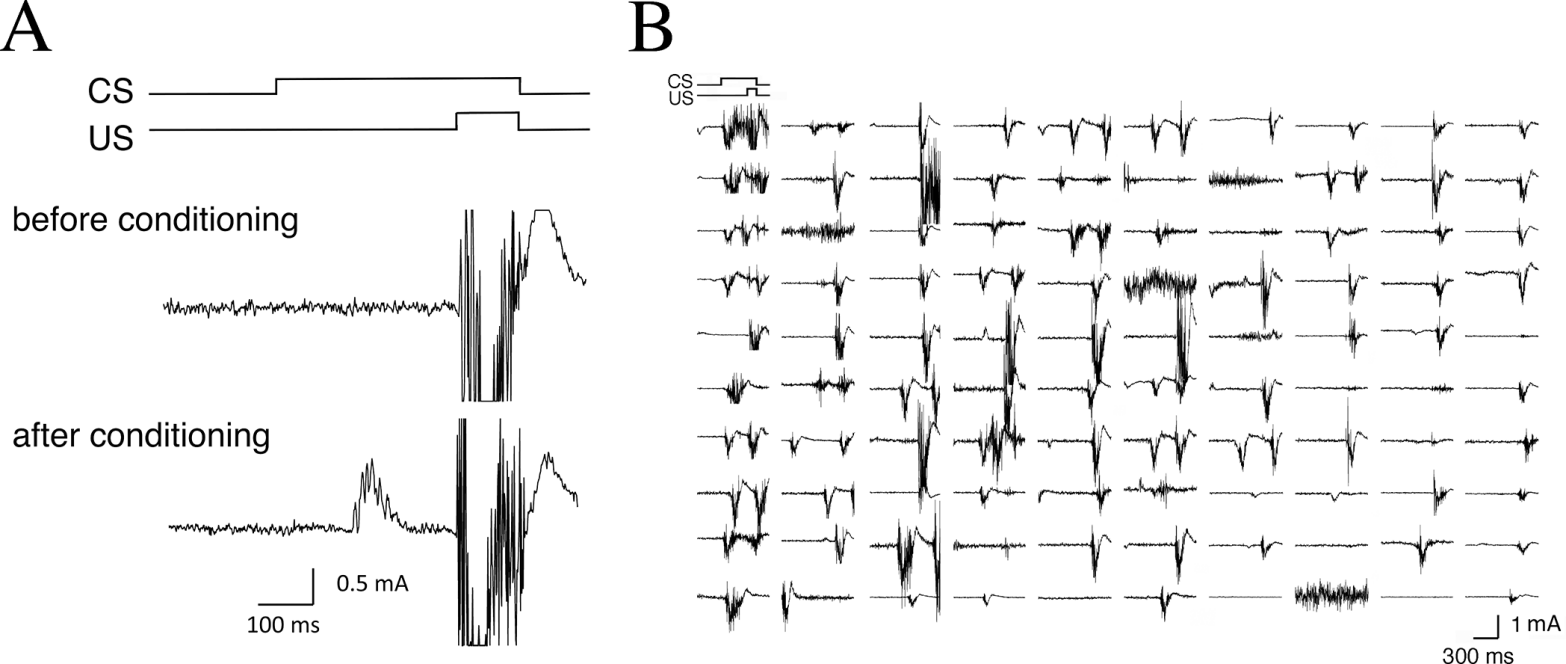
Examples of eyelid EMG responses in monkeys. (A) Typical EMG recordings are shown in the CS-US timing diagram. Before conditioning, the conditioned response (CR) is not observed (upper panel). However, repeated paired presentation of the CS and US results during expression of significant CR before US onset (lower panel). (B) All EMG responses from the 100 trials of 1 typical session on day 5 are shown.

We examined delay conditioned eyeblink responses in monkeys with OO-EMG measurement methods. Fig 2A shows typical EMG recordings from monkeys during the CS-US presentations. Although CRs could not be elicited before conditioning, repeated paired presentation of the CS and US induced obvious CR expression before US onset. Fig 2B shows all EMG data from one monkey from 1 day (100 trials on day 5).

**Fig 3:**
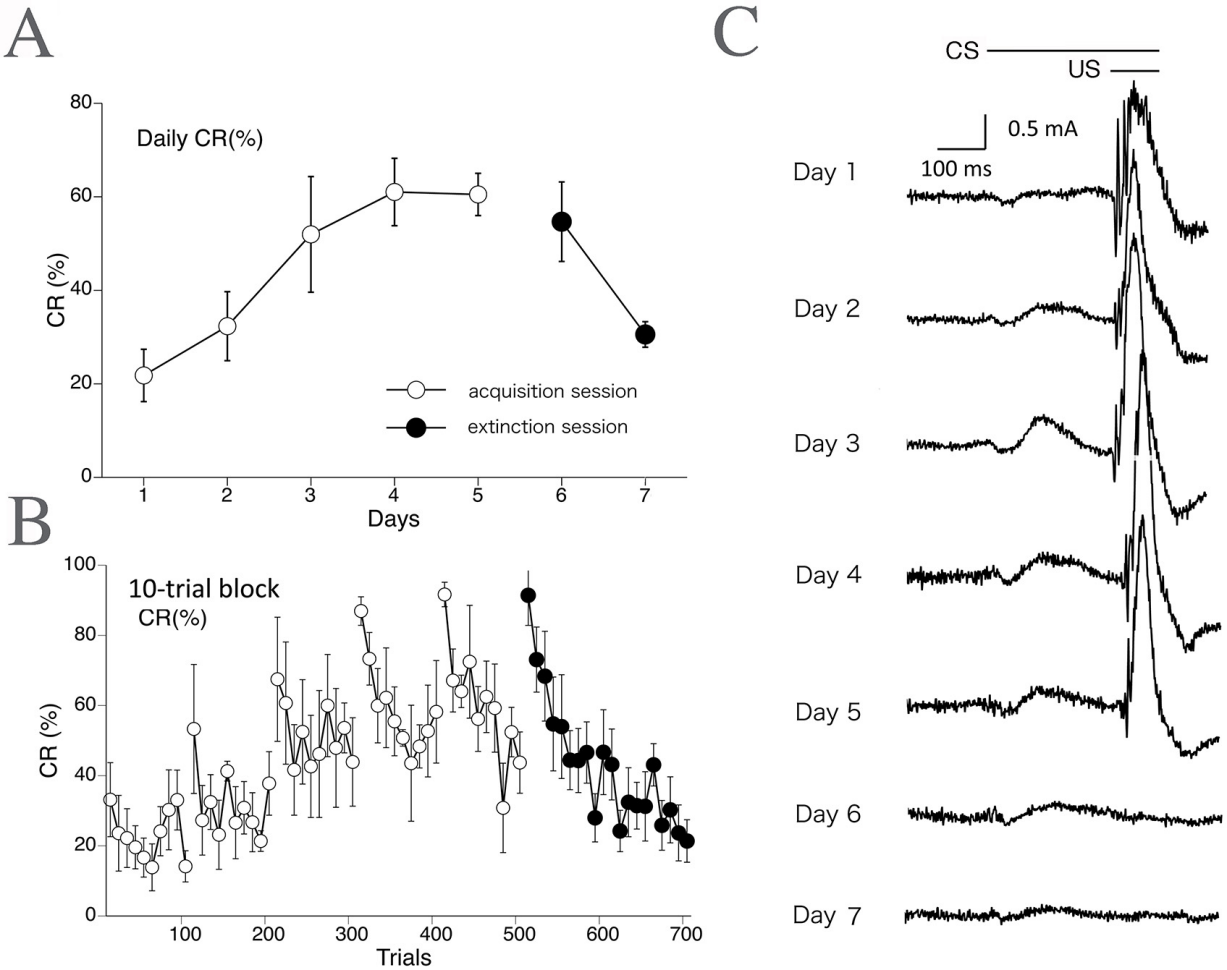
Delay eyeblink CR incidence (%) calculated across trials in monkeys (EMG data). The CR incidence (CR%) for all monkeys (*n* = 5) calculated on a daily (A) and 10-trial-block (B) basis, with 10 such blocks being run per day. (C) The rectified and averaged EMG amplitudes from all monkeys on days 1–7. In the extinction sessions, presentation of the CS without the US results in loss of the CR.

Fig 3A shows the learning curve (daily CR%) for the delay eyeblink conditioning averaged over all monkeys (*n* = 5). The CR% increased progressively up to 60.5% during the 5-day acquisition phase. Following the acquisition phase, the CR% decreased to 30.6% by the end of the 2-day extinction phase. Fig 3B shows the development of CR% calculated on every 10-trial block, showing within-session variations. CR% tended to be higher in the first 10 trials than in the last 10 trials during both the acquisition and extinction phases. Fig 3C shows the rectified and averaged EMG waveform on days 1–7, clearly indicating a gradual increase and decrease of the CR component over the acquisition and extinction sessions, respectively.

### Delay eyeblink conditioning in monkeys by high-speed video analysis

**Fig 4:**
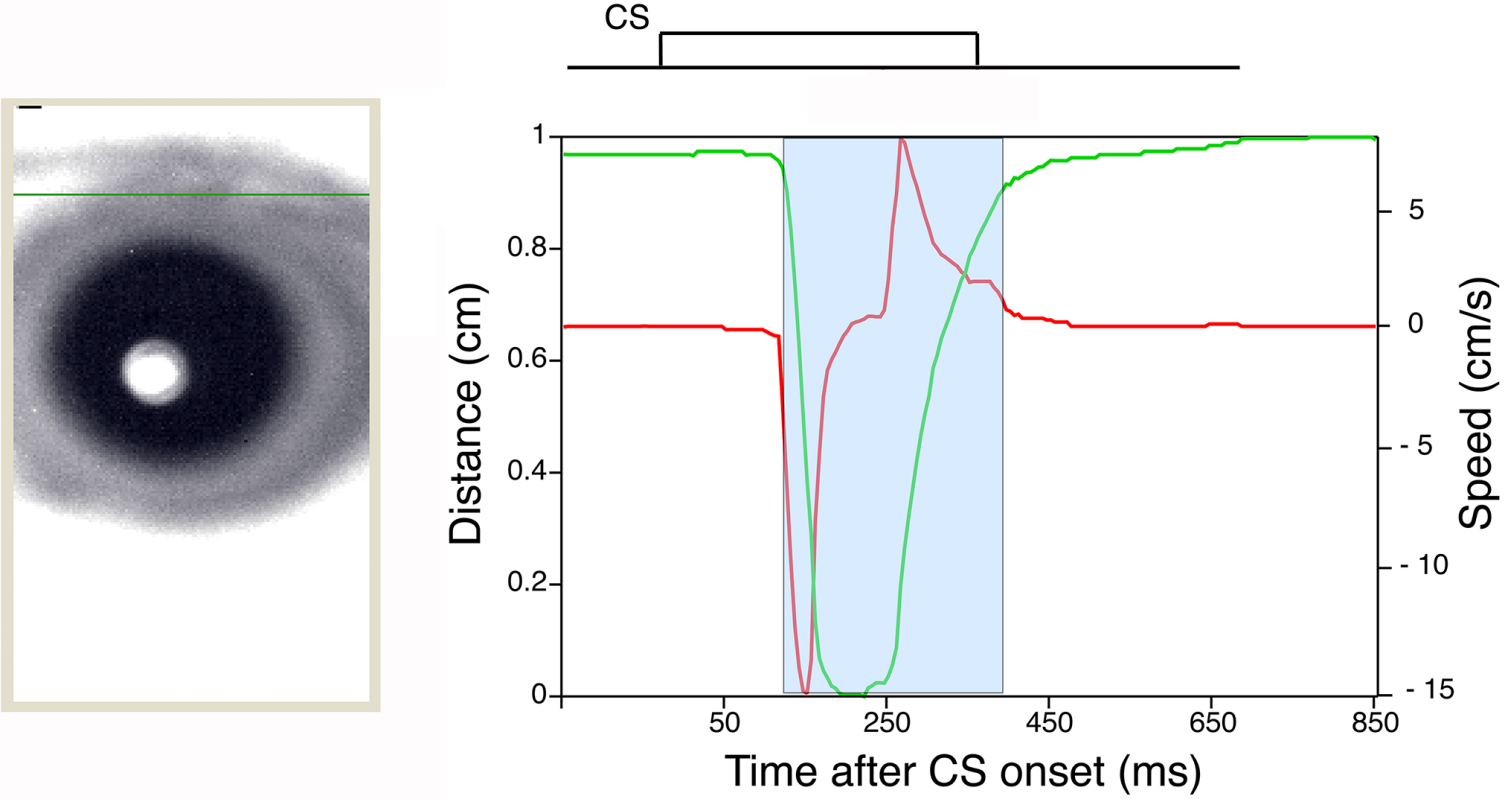
An example of eyelid movement obtained by high-speed video analysis. The eyelid movement data collected in a single CS-only trial after analysis with the Intelligent Vision Sensor. The green line plots the distance between the upper and lower eyelids versus time. The maximum eyelid distance (about 1 cm) represents the highest position of the upper eyelid (i.e., a completely open eye). Conversely, the status of the naturally close eyelid is defined as a distance of zero. The red line plots the velocity of the upper eyelid versus time. The blue area marks the period of time when the eyelid was moving, and the response within this area includes the CR. The left image showing the monkey’s eye represents the state of the eyelid immediately before CS onset. A green line was traced to mark the upper eyelid position. A schematic representation of CS timing for the delay eyeblink conditioning paradigm is shown above the plots of eyelid position and velocity.

Delay eyeblink conditioning in monkeys was next evaluated with a novel high-speed camera-based tracking system. Fig 4 shows an example of monkey eyelid movement analyzed with the high-speed camera-based tracking system (Fig 1A). The green solid line in Fig 4 shows an example of the position of the upper eyelid calculated as described in the Methods section.

**Fig 5:**
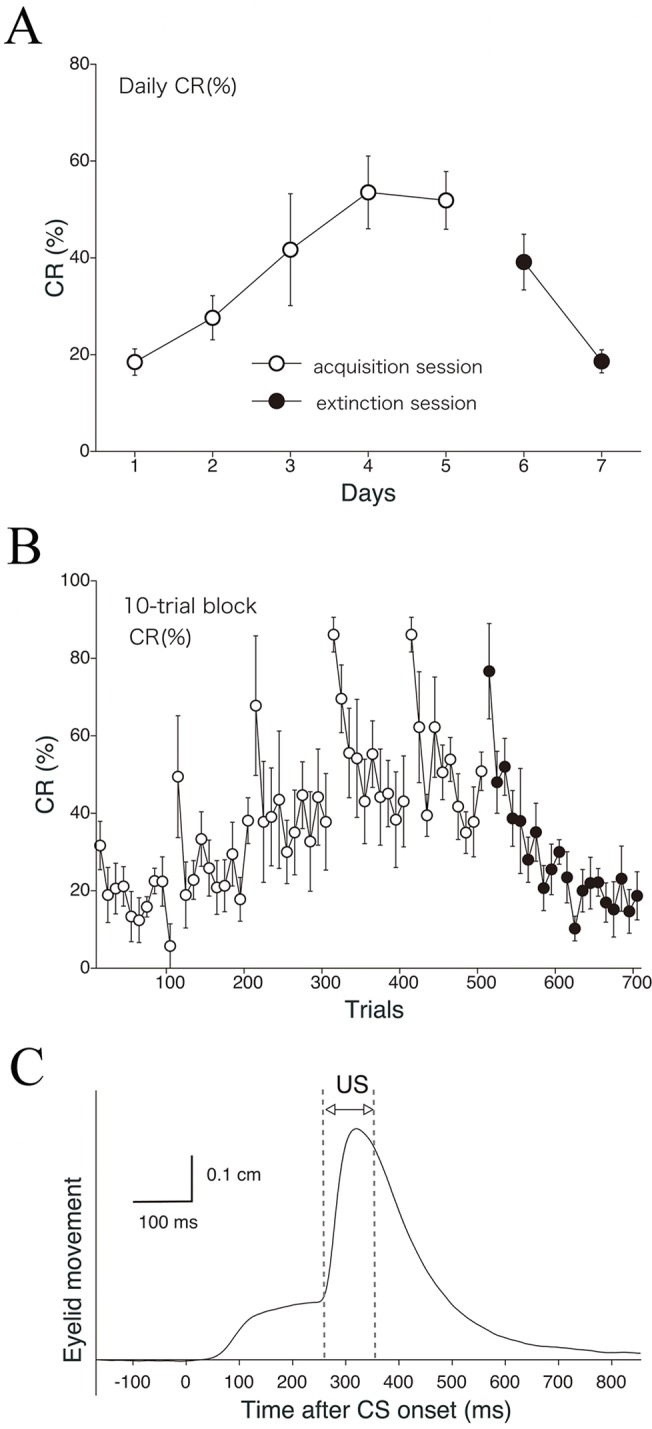
Delay eyeblink CR% in monkeys obtained by high-speed video analysis. The CR incidence (CR%) for all monkeys (*n* = 5) is calculated on a daily (A) and 10-trial-block (B) basis with data from the Intelligent Vision Sensor. Monkeys received five acquisition and two extinction sessions. (C) A plot of eyelid displacement versus time during a session (average of all monkeys on day 5).

Fig 5A depicts the day-by-day development of CR% as analyzed by the high-speed camera-based tracking system (*n* = 5). The CR% progressively increased up to 53.5% during the 5-day series of acquisition sessions. In the extinction sessions, CR% decreased to 18.6% in 2 days. Fig 5B shows the development of CR% calculated for every 10-trial block. The result shows intraday variation of CR%, indicating a tendency for a higher CR% in the first 10 trials compared to the last 10 trials during both the acquisition and extinction phases, as in the case of EMG-based analysis (Fig 3C). Fig 5C shows the averaged eyelid movement distance against time within a session (100 trials) for all monkeys (*n* = 5), indicating an obvious CR component before US onset.

### Comparison of CR detection sensitivity between EMG analysis and high-speed video sensing

**Fig 6:**
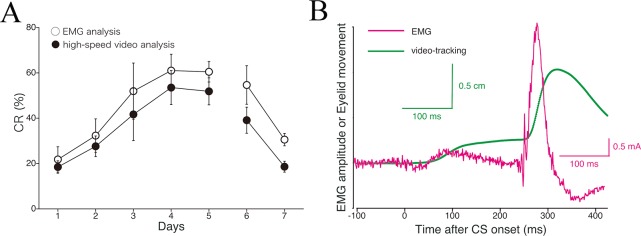
A comparison of the sensitivity of CR detection between EMG analysis and high-speed video analysis. (A) The all-subjects (*n* = 5) day-by-day average CR% is compared between the EMG and video-tracking channels. (B) Comparison of CR response timing analyzed by these two methods. The averaged EMG amplitude and video-recorded movement of the eyelid are compared in a single graph.

We next compared CR detection sensitivities between EMG analysis (Figs 2 and 3) and high-speed video analysis (Figs 4 and 5). In Fig 6A, the daily CR% as calculated by EMG analysis was compared with that determined with the high-speed video analysis. The EMG-based analysis showed that the monkeys exhibited successful CR acquisition during 5 days of acquisition sessions, and rapid extinction of CRs during 2 subsequent days of extinction sessions. Data from the high-speed video analysis showed the monkeys exhibiting similar tendencies during both CR acquisition and extinction. However, in the acquisition phase, CR% detected by EMG-based analysis was somewhat higher than that by high-speed video analysis, even though ANOVA revealed no significant difference in the CR acquisition results calculated from these two channels (*F*_1,8_ = 0.55, *p* = 0.48 for methodological effect; *F*_4,32_ = 0.24, *p* = 0.92 for interaction effect). The same trend favoring the EMG channel appeared in the extinction phase (*F*_1,8_ = 4.59, *p* = 0.064 for methodological effect; *F*_1,8_ = 0.18, *p* = 0.68 for interaction effect). Thus, EMG-based analysis is somewhat more sensitive for detecting eyeblink CR in monkeys. In Fig 6B, we compared the results of the two different principal methods from the viewpoint of CR topographies. The averaged EMG waveform and average position of the eyelid as a time function were plotted in a single graph. The CR peak given by the EMG method occurred at an earlier time than that given by high-speed video analysis.

### CR expression despite closed eyes

**Fig 7:**
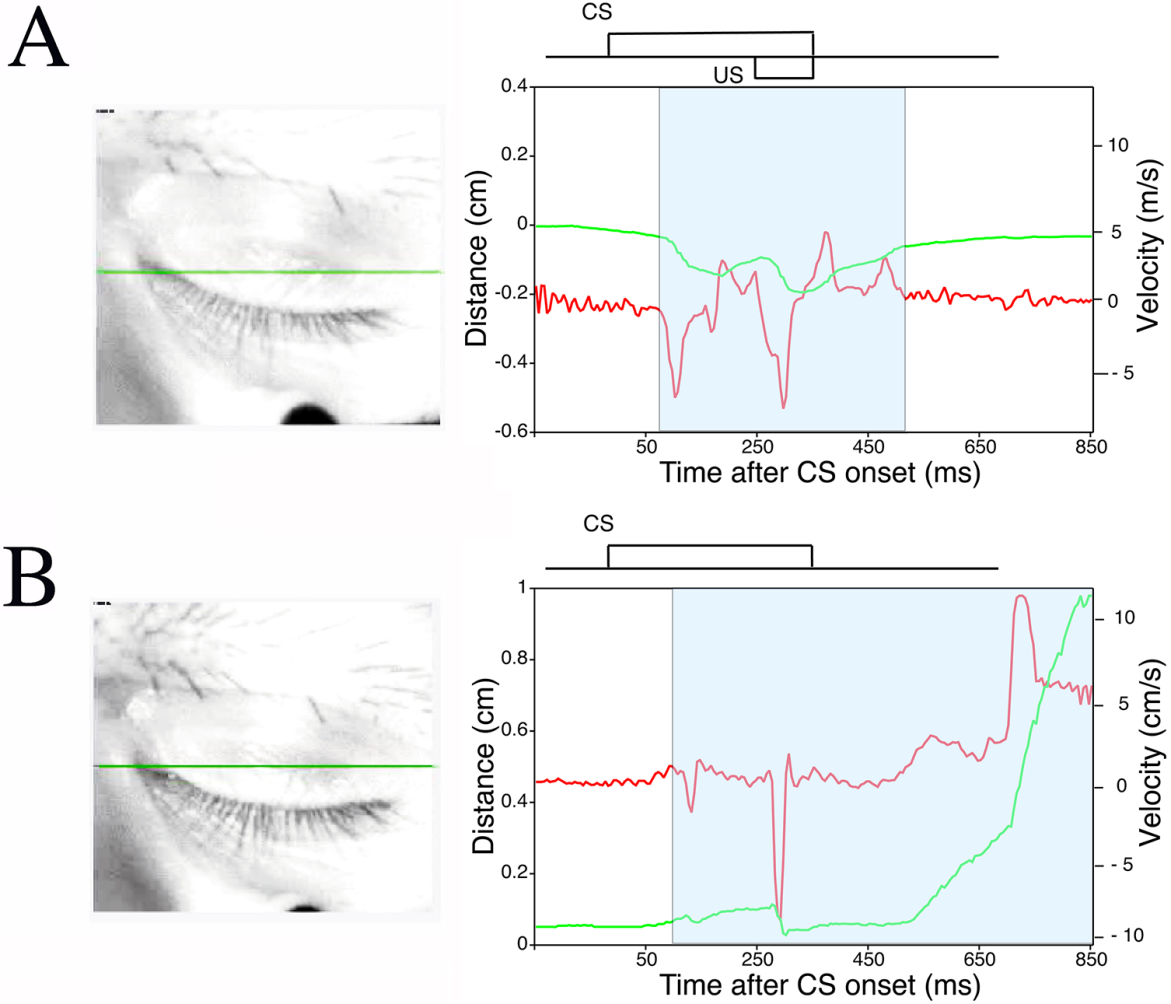
A preliminary study of CR expression during eyes-closed periods. Examples of CR expression during eyes-closed periods in a CS-US paired trial (A) and CS-only trial (B). Monkeys in light sleep often exhibit delay CRs despite their eyelids being completely closed (S1 and S2 Videos). Note that in such cases, the monkeys exhibit more downward movement of the upper eyelid than is needed to close the eye, resulting in a negative distance measurement. A schematic representation of the CS and US is inset above the plots of eyelid movement and speed. Green and red lines show the position and velocity of the upper eyelid, respectively. The red arrow indicates robust CRs between CS and US onsets.

**Fig 8:**
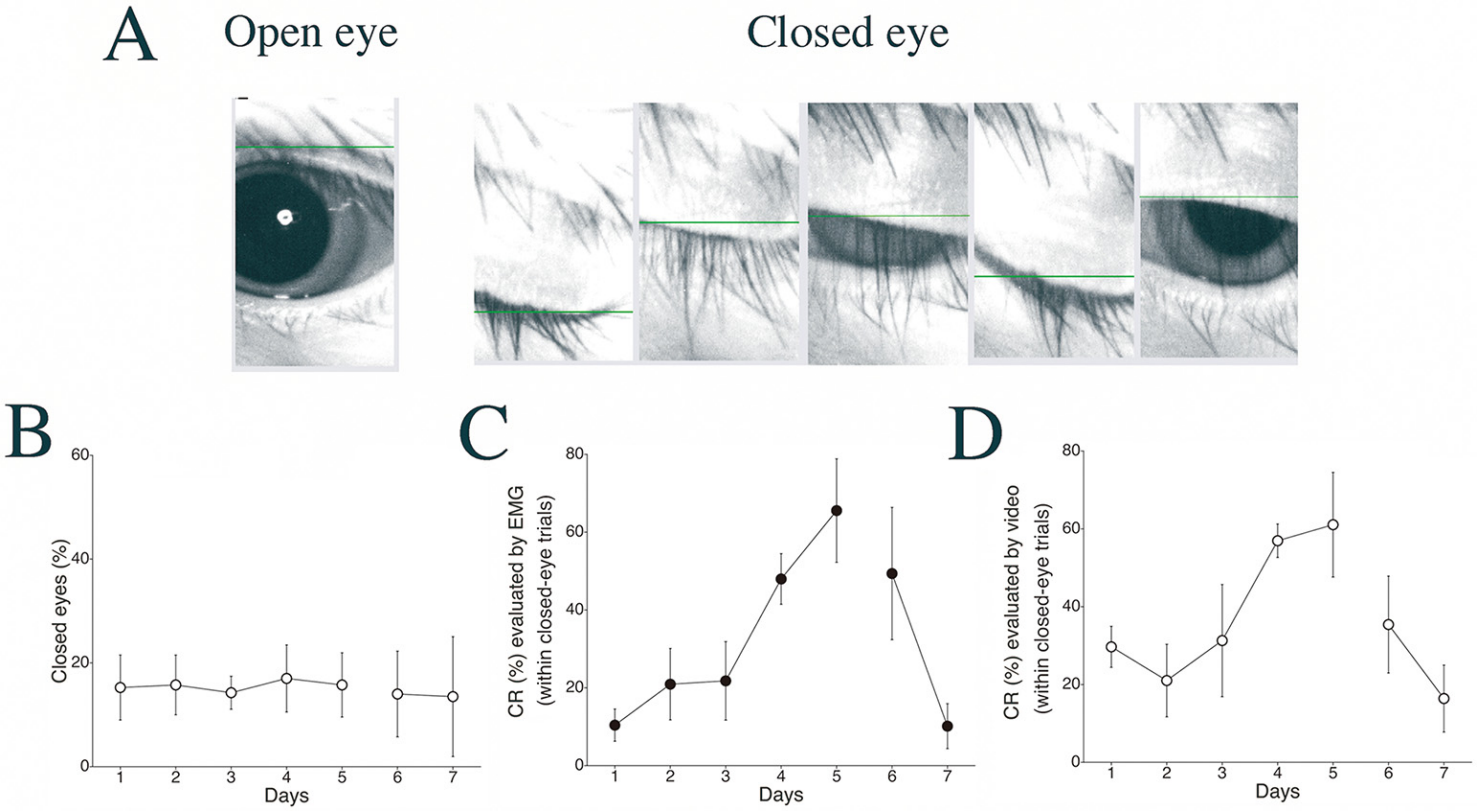
Changes in CR expression in cases of closed eyes. (A) Representative images of an “open eye” and a “closed eye” as judged by the video-tracking system. (B) The percentage of all trials that were “closed-eye trials” during delay eyeblink conditioning in monkeys. (C, D) The CR incidence (CR%) in the “closed-eye trials” as detected by EMG analysis (C) and as detected by the Intelligent Vision Sensor (D). Data are presented as mean ± S.E.M.

During conditioning, eye closures indicated that the monkeys often showed visible signs of drowsiness and behavioral microsleep. Rousing the subjects in such a situation would have introduced unacceptable artifacts into the data. Furthermore, in the process of assessing the relationship between the actual state of eyelid closure and OO-EMG activity, we found that even when their eyes were nearly closed, the monkeys frequently showed obvious CRs that were seen as a slight, further closure (Fig 7A and 7B and S1 and S2 Videos). S1 and S2 Videos show eyelid movement during a CS-US paired trial or a CS-only trial with obvious CRs during eyelid closure. Accordingly, we quantitatively evaluated the correlation between the actual state of eyelid closure (Fig 8A) and CR expression.

First, we calculated the incidence of conditioning trials in which the subject’s eye was closed just before CS onset (closed-eye trial, Fig 8B). Throughout the entire experimental period, the ratio of closed-eye trials to all trials (100 trials) held steady between 15% and 20%. Second, we calculated the CR incidence in the closed-eye trials by EMG analysis (Fig 8C). The results showed a significant CR% increase during the 5-day acquisition phase and a significant CR% decrease during the 2-day extinction phase. Interestingly, even in the high-speed video analysis, the CR was expressed during the closed-eye trials, and the CR incidence in the closed-eye trials was significantly increased and decreased in the acquisition and extinction phases, respectively (Fig 8D).

**Fig 9:**
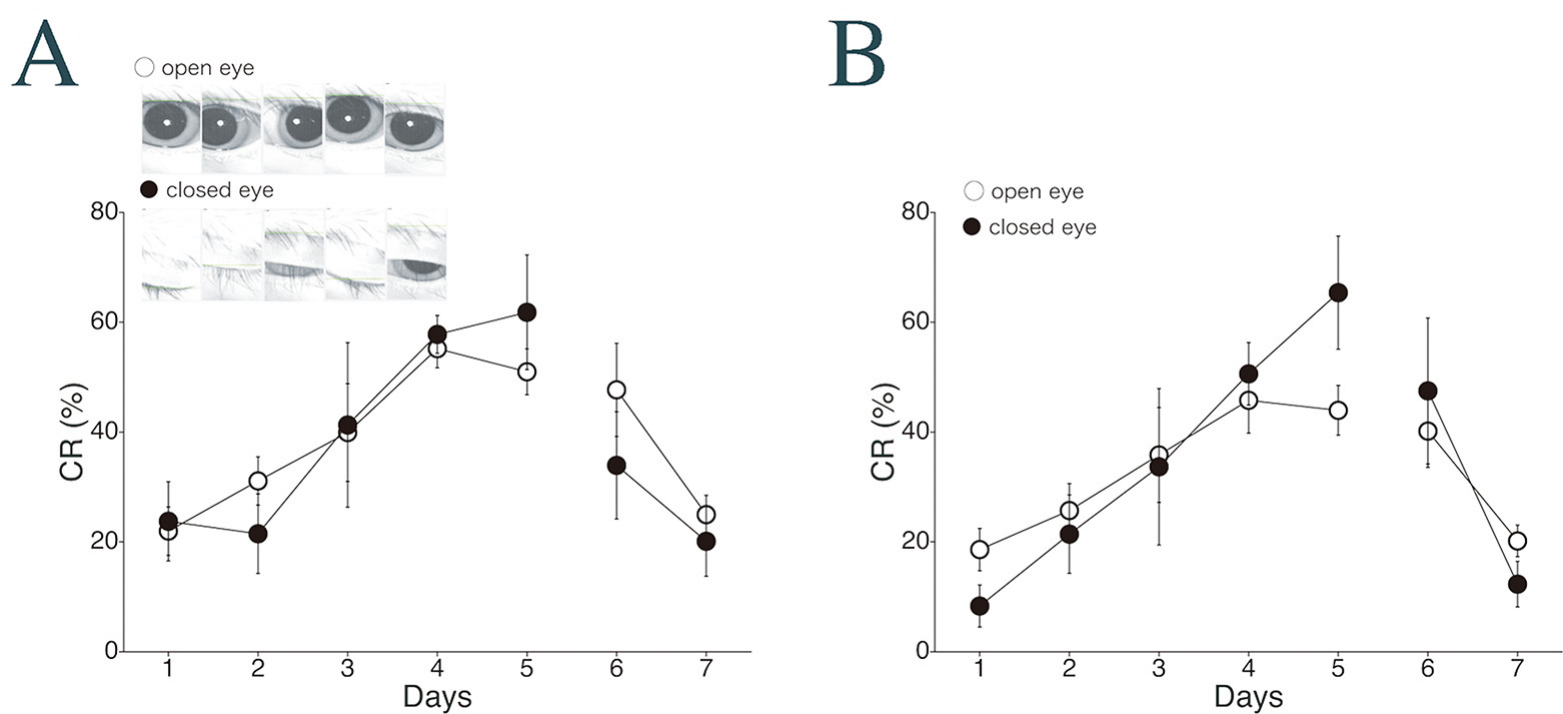
Changes in CR expression over the experiment in cases of open vs. closed eyes. The CR incidence (CR%) averaged over all monkeys (*n* = 5) in cases of eyes open and closed just before CS onset are compared between the EMG channel (A) and the Intelligent Vision Sensor channel (B). Insets in (A): representative images of an “open eye” and a “closed eye” as judged by the video-tracking system. Data are presented as mean ± S.E.M.

Third, to test the effect of eye-open/closed status on CR expression, we compared the CR expression incidence between closed- and open-eye trials (Fig 9). Using EMG-based analysis, no difference in CR% was found between closed- and open-eye trials during either acquisition (*F*_1,8_ = 0.028, *p* = 0.87 for eye-condition effect; *F*_4,32_ = 0.57, *p* = 0.69 for interaction effect) or extinction (*F*_1,8_ = 0.49, *p* = 0.51 for eye-condition effect; *F*_1,8_ = 1.18, *p* = 0.31 for interaction effect; see Fig 9A). Fig 9B shows the corresponding results obtained using video-based analysis. As with EMG analysis, no difference was detected between the closed- and open-eye trials during either acquisition (*F*_1,8_ = 0.060, *p* = 0.81 for eye-condition effect; *F*_4,32_ = 2.17, *p* = 0.095 for interaction effect) or extinction (*F*_1,8_ = 0.040, *p* = 0.084 for eye-condition effect; *F*_1,8_ = 0.88, *p* = 0.38 for interaction effect). These results show that delay CR incidence is not influenced by eyelid closure just before CS onset.

## Discussion

In the present study, we developed a monkey eyeblink conditioning system that simultaneously used an EMG method and 1-kHz high-speed camera-based tracking system [20, 28]. Although a measuring system for trace eyeblink conditioning in monkeys based on an infrared camera has already been developed [16], the time and spatial resolutions were poor. Compared with trace eyeblink conditioning, delay eyeblink conditioning studies require greater time resolution because the interval between CS and US onsets is quite short. In the present study, the high-speed camera-based tracking and EMG analysis systems—both sampling at 1 kHz—enabled us to analyze the detailed incidence and latency of eyeblink CRs in monkeys. Furthermore, because our system is a multi-measuring system that incorporates two different measuring principles, we were able for the first time to elucidate the relationship between the actual state of eyelid closure and OO-EMG activity in monkeys.

Using this novel system to record delay eyeblink conditioning in monkeys, we demonstrated successful CR acquisition and extinction (Figs 3 and 5). Even though the attained level of CR% (approximately 60%) in the present study is lower than that observed in cats and rabbits [13, 30, 31], it is comparable to or higher than the level reported in some human or mouse conditioning experiments [5, 12, 14, 24]. The present study is the first attempt to evaluate eyeblink conditioning in monkeys and is significant in three ways: as the first report of monkey eyeblink conditioning by EMG, as the first by video processing, and as the first report of the delayed version of eyeblink conditioning in monkeys. The monkey eyeblink CRs detected by the high-speed camera-based tracking system were in good agreement with those detected by EMG, although the EMG-based analysis gave a percentage of trials with observed CRs that was somewhat higher than that measured by high-speed video analysis (Fig 6). EMG analysis occasionally detected a CR in a trial when the high-speed camera-based tracking system did not. The opposite also occurred, but with a lower incidence. Latency analysis showed that the CR peak for the EMG method preceded that for high-speed video analysis (Fig 6B). This is consistent with previous studies in other species and likely reflect the fact that the muscle EMG induces physical eyelid movement [32, 33]. A detailed analysis also indicated that CR incidence tended to be higher in the first half of trials than in the latter half during each daily session (Figs 3B and 5B). This intraday variation in CR occurrence is comparable to that observed in mice [18]. The phenomenon is probably attributable to habituation to the stimulus or testing environment.

In the present conditioning procedure, each animal was placed in a monkey chair in a dimly lit room for more than 30 min every day. Thus, it often appeared that the monkeys dozed during conditioning, but we did not wake them up in such situations. The multi-measuring system could detect the actual state of eyelid closure and the monkey’s appearance, so we could separately evaluate performance in awake and light sleep states. As shown in S1 and S2 Videos, an interesting subsidiary finding was that the monkeys often exhibited obvious CRs even when their eyes had closed just before CS onset (Fig 7). Quantitative analysis confirmed that the probability of CR incidence was not influenced by whether or not the monkeys closed their eyelids just before CS onset. In fact, the incidence of both eyes-open and eyes-closed CRs rose and fell in parallel over the acquisition/extinction sequence (Fig 9). These results exclude the possibility that CR responses during closed-eye trials were startle responses caused by the tone CS.

Delay eyeblink conditioning has been tested in many animal species including human subjects, but few studies have evaluated this paradigm in monkeys [12, 13]. This is somewhat surprising considering that one other major paradigm of cerebellar motor learning, the vestibulo-ocular reflex (VOR), has been extensively performed in monkeys [34, 35]. Clark and Zola (1998) suggested several reasons for the absence of contemporary studies of eyeblink conditioning in monkeys prior to their research [16], though several premodern attempts had been made [36–38]. One of the reasons was that the expense of primate models is only justified by their special relevance to the cognitive aspects of human memory. Thus, it could be supposed that there was no perceived need to develop an eyeblink conditioning system for monkeys to supplement research on simple forms of human learning that are not especially evolved in humans, such as eyeblink conditioning [16]. However, the context of monkey biology has greatly changed in recent years. Monkey research is embarking upon a new phase because of the development of genetically engineered nonhuman primates [39–41]. In the new era, the monkey eyeblink conditioning system could be a meaningful tool for elucidating the molecular basis of implicit and explicit memory [5, 42, 43]. Moreover, a positron-emission tomographic (PET) method for measuring brain functions in monkeys in a conscious state allows us to map brain functions and determine which regions are activated when the higher functions that are involved in learning, memory, or cognition are exercised [44, 45]. In future, the combination of the conscious, nonanesthetized preparation with PET imaging and monkey eyeblink conditioning technology may more clearly elucidate the neural system(s) underlying delay eyeblink conditioning in higher primates, thus enabling direct, subtractive comparison with systems underlying higher functions.

The neural circuit involved in eyeblink conditioning is now considered well defined, although several unsolved questions remain concerning the relative roles of the cerebellar cortex and cerebellar deep nuclei [46, 47] even though several studies have assumed a different role of the cerebellum in this type of associative learning [30, 48]. At least in rabbits and rodents, delay conditioning is critically dependent on a brainstem-cerebellar circuit [4, 8, 46, 49–51], whereas trace conditioning with a sufficiently long stimulus-free interval depends on the condition of the hippocampus [8]. Moreover, substantial research in humans suggests the importance of the cerebellum in the memory of delay eyeblink conditioning, which is consistent with the small-animal data [52–55]. However, a species difference in brain regions critical for delay-eyeblink memory formation may exist between higher mammals (including humans) and rodents. For example, in humans more than in rodents, the hippocampal contribution may be greater in the acquisition of delay eyeblink memory because delay eyeblink conditioning is a more sensitive test for cognitive deficit in patients with Alzheimer disease (AD) than the trace eyeblink conditioning paradigm [56], despite the fact that the cerebellum is a relatively spared area of the AD brain and that general motor learning is preserved in these patients [57, 58]. In contrast, AD model mice exhibited significant impairment in long-trace but not delay eyeblink conditioning [23, 25, 59]. Delay eyeblink conditioning in monkeys could prove useful in identifying the brain regions that are critical for motor memory in higher mammals. An important issue is whether primates and rodents share common brain regions for implicit memory.

The observation of learning expressed as a behavioral output during microsleep or drowsiness is quite interesting and rare. Our present results suggest that delay eyeblink memory can be expressed and might even be formed at low levels of arousal. This is consistent with the notion that delay eyeblink conditioning is a form of nondeclarative implicit memory [3]. Our present results from a novel multi-measuring system for eyeblink CRs in monkeys have important implications for elucidating the neural mechanisms underlying implicit/unconscious learning.

In conclusion, we have developed the first monkey eyeblink conditioning system using simultaneous EMG and high-speed video analysis. The monkey eyeblink CRs detected by the high-speed camera-based tracking system were in good agreement with those detected by EMG analysis. This multi-measuring system incorporating two different measuring principles enabled us to elucidate the relationship between the actual presence of eyelid closure and OO-EMG activity during the experiment. Interestingly, the animal’s eyelid-open state, which is supposed to be related to the animal’s wakefulness and potential presence of cognitive processing [29], did not predict the probability of CR incidence. These results suggest that delay eyeblink memory can be expressed independently of behavioral state (degree of wakefulness or consciousness), confirming that this form of conditioning is implicit learning. An interesting problem for future studies is to determine whether the CR is observed in monkeys during trace eyeblink conditioning, which is characterized by a brief stimulus-free period between CS offset and US onset. Our system for cognitive assessment in monkeys based on eye closure will be a useful tool for elucidating the neural mechanisms underlying delay eyeblink conditioning—an acquisition process for implicit memory in higher mammals.

## Supporting Information

S1 Video Example of eyelid movement during a CS-US paired trial. An obvious CR is elicited before US onset even during closed-eye periods. Left: The physical movement of the eyelid is tracked by the Intelligent Vision Sensor; the green horizontal line illustrates the position of the upper eyelid over time. Right: time course of eyelid movement. The green line shows the position of the upper eyelid, and the red line shows velocity. The scale of the vertical axis is inverted relative to Fig 7A. The relationship between the CS and US is depicted above the time courses of eyelid movement.
(MP4)

S2 Video Example of eyelid movement during a CS-only trial. An obvious CR is elicited before US onset even during closed-eye periods. Left: The physical movement of the eyelid is tracked by the Intelligent Vision Sensor; the green horizontal line illustrates the position of the upper eyelid over time. Right: time course of eyelid movement. The green line shows the position of the upper eyelid, and the red line shows velocity. The CS timing is depicted above the time courses of eyelid movement.
(MP4)
